# Tandem and cryptic amino acid repeats accumulate in disordered regions of proteins

**DOI:** 10.1186/gb-2009-10-6-r59

**Published:** 2009-06-01

**Authors:** Michelle Simon, John M Hancock

**Affiliations:** 1 Bioinformatics Group, MRC Harwell, Mammalian Genetics Unit, Harwell Science and Innovation Campus, Harwell, Oxfordshire, OX11 0RD, UK

## Abstract

**Background:**

Amino acid repeats (AARs) are common features of protein sequences. They often evolve rapidly and are involved in a number of human diseases. They also show significant associations with particular Gene Ontology (GO) functional categories, particularly transcription, suggesting they play some role in protein function. It has been suggested recently that AARs play a significant role in the evolution of intrinsically unstructured regions (IURs) of proteins. We investigate the relationship between AAR frequency and evolution and their localization within proteins based on a set of 5,815 orthologous proteins from four mammalian (human, chimpanzee, mouse and rat) and a bird (chicken) genome. We consider two classes of AAR (tandem repeats and cryptic repeats: regions of proteins containing overrepresentations of short amino acid repeats).

**Results:**

Mammals show very similar repeat frequencies but chicken shows lower frequencies of many of the cryptic repeats common in mammals. Regions flanking tandem AARs evolve more rapidly than the rest of the protein containing the repeat and this phenomenon is more pronounced for non-conserved repeats than for conserved ones. GO associations are similar to those previously described for the mammals, but chicken cryptic repeats show fewer significant associations. Comparing the overlaps of AARs with IURs and protein domains showed that up to 96% of some AAR types are associated preferentially with IURs. However, no more than 15% of IURs contained an AAR.

**Conclusions:**

Their location within IURs explains many of the evolutionary properties of AARs. Further study is needed on the types of IURs containing AARs.

## Abstract 2

Analysis of amino acid repeats in four mammalian and one bird genome shows that many are associated preferentially with intrinsically unstructured regions.

## Background

Amino acid repeats (AARs) are segments of proteins made up of simple patterns of amino acids, often strings of a single amino acid. They have long been recognized to be common features of eukaryotic proteins [1-4]. Polyglutamine repeats, the most intensively studied class because of their association with human diseases such as Huntington's [5], tend to be evolutionarily labile, especially when encoded by pure repeats of the codon CAG [6,7]. Because of this lability, AARs have often been considered to be evolutionarily neutral structures [8]. However, a number of experimental studies [9-12] suggest that AARs play an important role in protein function. Studies of the functions of AAR-containing proteins also suggest that they are preferentially found within certain classes of proteins. From the earliest reports through to the most recent genome-wide surveys in *Saccharomyces cerevisiae *[3,13,14] and mammals [15] a consistent pattern of association with transcription has emerged for the most common tandem repeat types. Additional associations, notably with protein kinases [13], suggest possible involvement in cellular signaling networks, which in turn suggest that repeats could play a significant role in the evolution of such networks [16]. Finally, studies of the relationship between morphology and repeat length in dog breeds [17] have shown that variation at repeat loci can have evolutionarily significant effects on phenotype. Polyalanine repeats have also been found to be involved in a number of genetic diseases, in this case involving developmental defects [18]. Removing a polyalanine tract from murine Hoxd-13 has a direct effect on bone phenotype [19], again indicating involvement of an AAR in an important biological process.

AAR size difference between orthologous human and mouse proteins correlates with protein nonsynonymous substitution rate [20]. A study of the factors contributing to the evolutionary expansion of polyglutamine repeats in a limited number of human-mouse orthologues [21] concluded that labile repeats, which are encoded by homogeneous runs of a single codon [6], have a strong tendency to arise in regions of proteins subject to weaker purifying selection than the protein as a whole, while repeats that are more conserved did not show this tendency. This has been supported recently by a large-scale study of human, mouse and rat repeats [22]. These observations suggest a model for repeat evolution whereby initially labile repeats become fixed when they reach some optimal length range [21]. Human polyglutamine disease genes might then be still evolving towards such an optimum.

Intrinsically unstructured regions (IURs), also called disordered regions, are regions of protein, ranging in size from short loops to complete proteins, that do not form a compact tertiary structure under normal solvation conditions [23]. They have been suggested to be involved in protein-ligand binding, including protein-protein interactions, forming compact structures only when bound to a cognate ligand [24]. Tompa [25] pointed out that many IURs contain AARs and suggested that IURs may evolve to a considerable extent by the expansion of such repeats. Disordered proteins - that is, proteins primarily made up of IURs - have also been suggested to have lower sequence complexity than ordered proteins [26]. Tompa's suggestion [25] would be consistent with the relatively rapid sequence evolution of many IURs [27,28], the observation that highly connected (hub) proteins in protein interaction networks appear to be enriched in AARs and in proteins containing IURs [29], and the suggestion that evolution of AARs could have an effect on network evolution by altering protein-protein affinities [16]. As Tompa [25] analyzed only a relatively small set of IURs, his hypothesis raises the question whether AARs show a preferential location in IURs, and whether any such preference could account for the evolutionary properties of the bulk of AARs in a proteome. Such a preference would be consistent with hypotheses on the causation of triplet expansion diseases that invoke destabilization of protein structure as an important causative factor [18].

A variety of computational methods exist to detect repeated sequences in proteins. These range from SEG, which looks for regions of low complexity [30], to alignment-based approaches [31]. Here we use an extended definition of amino acid repetition that includes cryptic repeats as measured by the program SIMPLE, which we have previously used to look at AARs in the yeast proteome [32], as well as tandem AARs. This allows us to study repeats below the normal threshold taken for tandem repeats (five amino acids) and regions with significant biases in amino acid content that are not tandem in nature but may have originated from tandem repeats (C4 repeats; see Materials and methods for more detail).

Using a set of orthologues to human genes from four species (chimpanzee, mouse, rat and chicken; *Pan troglodytes*, *Mus musculus*, *Rattus norvegicus *and *Gallus gallus*) we show that the most common AARs show strong preferences to be located within IURs in all five proteomes. We also confirm that sequences flanking AARs evolve more rapidly than the remainder of their respective proteins. We conclude that the forces shaping the evolution of IURs and AARs are strongly linked, although AARs are present in only a subset of IURs.

## Results

### Repeat frequencies

**Figure 1:**
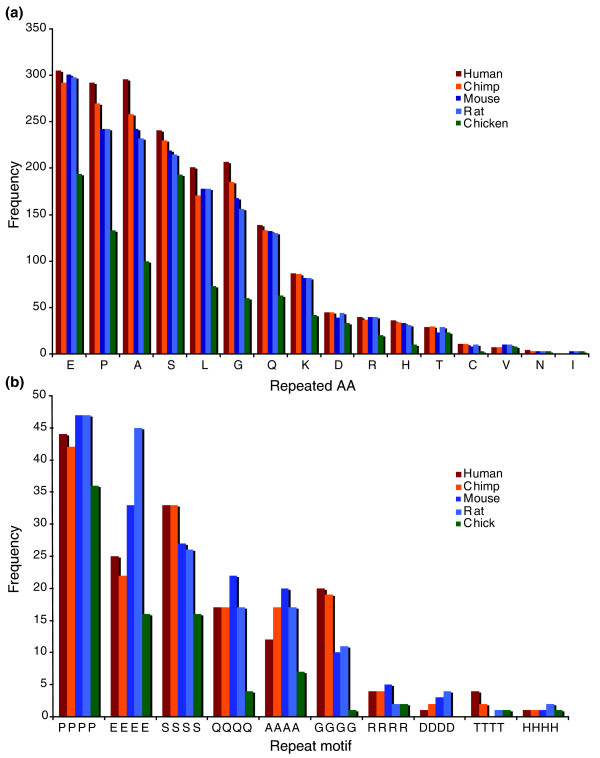
Frequencies of common AAR types in the five proteomes studied. **(a) **Absolute frequencies of all observed tandem amino acid types. Repeat types are ordered by mean frequency. Bars are color coded as follows: brown, human; orange, chimpanzee; dark blue, mouse; light blue, rat; green, chicken. **(b) **Frequencies of C4 tandem-like repeats making up more than 1% of the complement of C4 repeats. Color coding as for (a).

Our protein set contained 5,815 orthologous proteins. Figure 1 shows the frequencies of tandem and C4 cryptic repeats in this set: Figure 1a shows frequencies for all detected single amino acid repeats and Figure 1b shows frequencies for all C4 repeats with a homogeneous repeat motif (such as Q_4_). (Homogeneous C4 repeats are regions containing a significant overrepresentation of runs of a single amino acid of length 4; they therefore differ from tandem repeats of that amino acid because they fall below the definition of a tandem repeat. Throughout this paper, tandem repeats of an amino acid are referred to by the single letter code for the amino acid concerned. Homogeneous cryptic repeats are referred to as X_4 _repeats, where 'X' is the single letter code for the repeated amino acid.) It should be noted that numerous other non-homogeneous C4 motifs were detected; these are not considered here.

Comparing the frequencies of homogeneous C4 repeat types with their tandem equivalents showed significant correlations (*P *< 0.01 or less after Bonferroni correction) ranging from 0.555 (chicken) to 0.718 (rat). Despite this broad similarity it was noteworthy that L_4 _repeats were absent amongst C4 repeats, although relatively common among tandem repeats.

The frequency distributions of the tandem repeat types are highly similar between the four mammals, with correlation coefficients > 0.99 (*P *<< 0.001) for all six pairwise comparisons. The distribution for chicken correlates less well with those seen in mammals, showing correlation coefficients ranging from 0.894 (human-chicken) to 0.929 (rat-chicken). In general, chicken proteins contained fewer tandem repeats than mammalian proteins (961 in total, compared to 1,940, 1,792, 1,723 and 1,703 for human, chimpanzee, mouse and rat, respectively). Serine tandem repeats were less extreme in this respect, chicken proteins containing 193 repeats compared to 241, 230, 219 and 215 for the mammals.

We also calculated inter-species correlation coefficients between the frequencies of the commonest homogeneous C4 repeats. These C4 repeats also showed strong and significant (*P *<< 0.001) correlations between frequencies in all five species, ranging from 0.870 for chimpanzee-rat to 0.989 for human-chimpanzee. C4 repeats were rarer in chicken proteins than mammalian proteins, glycine (G_4_) and glutamine (Q_4_) C4 repeats being particularly underrepresented in chicken.

**Figure 2:**
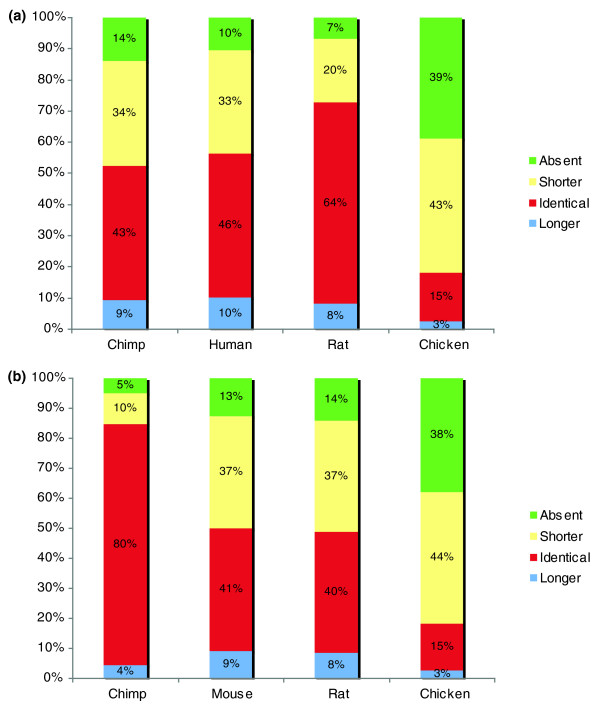
Conservation of tandem AARs from the perspective of the human and mouse protein sets. **(a) **Vertical bars represent proportions of tandem repeats that are absent (light blue), shorter in the target species (yellow), identical in the target species (red) or longer in the target species (purple). Target species (that is, species tested for presence or absence of human repeats) are ordered by phylogenetic closeness to human. **(b) **Corresponding plot for mouse repeats.

**Figure 3:**
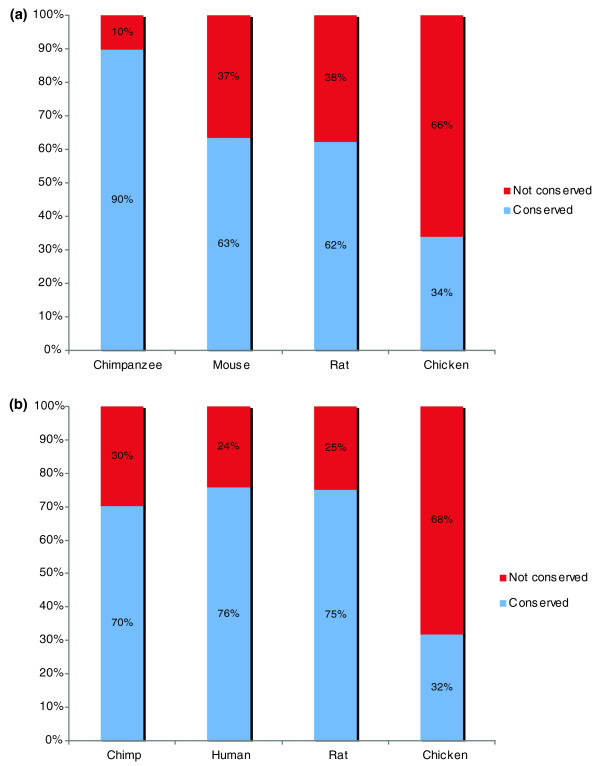
Conservation of C4 AARs from the perspective of the human and mouse protein sets. **(a) **Vertical bars represent proportions of tandem repeats that are present (purple) or absent (red). **(b) **Corresponding plot for mouse repeats.

Finally we considered the proportion of repeats conserved between pairs of species, as judged by the absence or presence of repeats at the same position in pairs of orthologs. This enabled us to classify repeats into conserved and non-conserved classes between any two species and provides a measure of the relative degree of conservation of tandem and C4 repeats. Figures 2 and 3 show the results of these analyses. Generally, conservation of both tandem and C4 repeats decreased with phylogenetic distance, as might be expected. This pattern was seen whether the repeats compared to other species were identified in human or mouse proteins.

### Evolutionary divergence

It has been suggested that regions surrounding tandem repeats are under weaker purifying selection than the remainder of the protein they are embedded in [7,21,22]. Recent evidence also suggests that repeat-containing proteins evolve more rapidly than non-repeat-containing proteins [33]. IURs, on average, also show more rapid evolution than the average protein [27]. To confirm that repeats are located in regions under relatively weak purifying selection we measured pairwise protein sequence distances between orthologues. Proteins were subdivided into those with conserved repeats (that is, present in both species) and non-conserved repeats (present in only one), as previous analyses suggested that only non-conserved repeats lie in regions of lower purifying selection [21].

Table 1 summarizes the results of these analyses. Sequences flanking both tandem and cryptic repeats evolve significantly more rapidly than the remainder of the protein they are part of. The difference between flanking sequence and protein remainder is larger for non-conserved repeats than conserved repeats but both show the effect. This is broadly consistent with previous observations based on a small set of conserved and non-conserved repeats [21], which showed elevated divergence around non-conserved but not conserved repeats. Divergences around conserved AARs were lower than those around non-conserved AARs, and conserved repeats tended to lie in more conserved proteins than non-conserved repeats.

**Table 1:** Mean divergences of repeat flanks versus protein remainder

	Tandem						C4					
		
	Conserved			Non-conserved			Conserved			Non-conserved		
				
Comparison	Flank	Rest	P*	Flank	Rest	P*	Flank	Rest	P*	Flank	Rest	P*
Human-mouse	0.152	0.074	8.2×10^-13^	0.352	0.129	3.7×10^-7^	0.151	0.082	2.7×10^-5^	0.394	0.219	2.6×10^-4^
Human-rat	0.175	0.083	7.5×10^-13^	0.345	0.130	1.6×10^-9^	0.151	0.076	6.5×10^-6^	0.435	0.218	3.8×10^-3^
Human-chicken	0.350	0.194	3.8×10^-5^	0.862	0.346	4×10^-19^	0.413	0.226	6.0×10^-4^	0.761	0.305	4.6×10^-10^

*P-value for flanking and remainder rates being different (two-tailed *t*-test). All differences are significant after Bonferroni correction.

To estimate more precisely the relative increase of evolutionary divergence in the neighborhood of repeats, we carried out regression analysis. The slope of the regression of the flanking sequence divergence on the corresponding protein remainder divergence represents the relative enhancement of flanking sequence divergence in a given dataset. Regression results for human-mouse, human-rat and human-chicken comparisons are summarized in Table 2. Non-conserved tandem repeats show more than twice the divergence in the neighborhood of repeats than in the remainder of the corresponding protein in human-rodent comparisons. This ratio is somewhat lower in the human-chicken comparison, possibly because of the effects of mutational saturation, which would have the effect of reducing the estimated divergence of the more rapidly evolving regions. For conserved tandem repeats the elevation was of the order of 50%, which is more modest but still significant. C4 repeats showed a weaker elevation of divergence rate, of the order of 10 to 15% for most human-rodent comparisons. The elevation for human-chicken comparisons was comparable to that seen for tandem repeats but was not statistically significant (*P *> 0.05 after Bonferroni correction).

**Table 2:** Regression results of repeat flank divergence on protein remainder divergence

	Tandem				C4			
		
	Conserved		Non-conserved		Conserved		Non-conserved	
				
Comparison	m*	P_r>1_^†^	m*	P_r>1_	m*	P_r>1_	m*	P_r>1_
Human-mouse	1.557	6.3×10^-7^	2.208	6.1×10^-4^	1.137	(0.607)	1.121	(0.051)
Human-rat	1.535	2.4×10^-7^	2.326	7.05×10^-7^	1.468	(0.070)	1.115	(0.289)
Human-chicken	1.448	8.1×10^-4^	1.623	1.1×10^-6^	1.679	(0.005)	1.890	(0.047)

*Slope of the regression line between the divergences of a sequence's flanking repeats and the rest of the protein. ^†^*P*-value for the slope of the regression line being greater than 1. *P*-values that are not significant after Bonferroni correction are in parentheses.

### Functional (Gene Ontology term) association

A number of authors have discussed associations of tandem and cryptic AARs with transcription factors and protein kinases in particular [1,3,13-15,34-36]. Here we consider the Gene Ontology (GO) term associations of repeat-containing members of our orthologue set in comparison with the rest of the set. We looked for significant associations (*P *< 0.05 after adjustment for false discovery rate) at levels 3 and 4 of the GO molecular function hierarchy. We carried out the analyses for human and chicken to characterize any differences reflected in the different repeat frequencies seen in the chicken and mammal proteomes.

**Figure 4:**
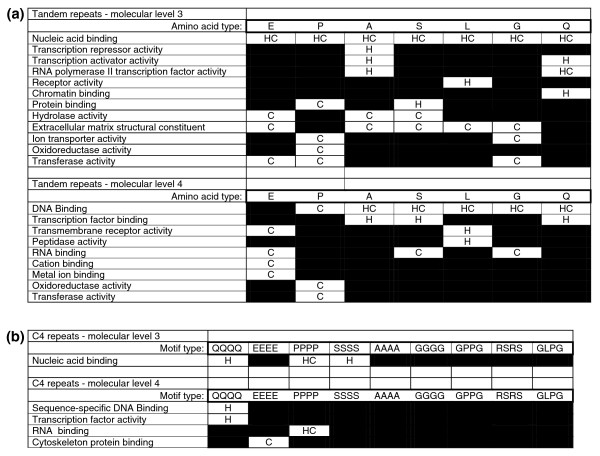
Overrepresented Gene Ontology terms in human or chicken proteins containing AARs. **(a) **Tandem repeats; **(b) **C4 repeats. Terms showing significant overrepresentation after correction for multiple testing are labeled according to the species in which overrepresentation was observed: H, human; C, chicken; HC, both. GO terms were tested for overrepresented at two levels: level 3 and level 4. The terms are separated by level in the figure.

Results were broadly similar to those obtained previously for yeast and other species [13,15] (Figure 4). All of the common tandem AAR types showed significant association with nucleic acid binding proteins in both human and chicken, and A, S, L, G and Q repeats also showed associations with DNA binding proteins in both species. Q repeats also showed a specific association with RNA polymerase II transcription. A number of other associations were seen in human or chicken but not both. The importance of these is unclear.

C4 repeats showed fewer common associations between the human and chicken proteins sets. The only shared association was found for P_4 _repeats with RNA binding (level 3: nucleic acid binding). In humans, Q_4 _repeats showed qualitatively similar associations to those seen for tandem Q repeats. E_4 _repeats also showed an association with cytoskeleton protein binding in chicken, which is to some extent similar to the cytoplasmic roles identified for tandem E repeats.

### Domain and intrinsically unstructured region associations

**Figure 5:**
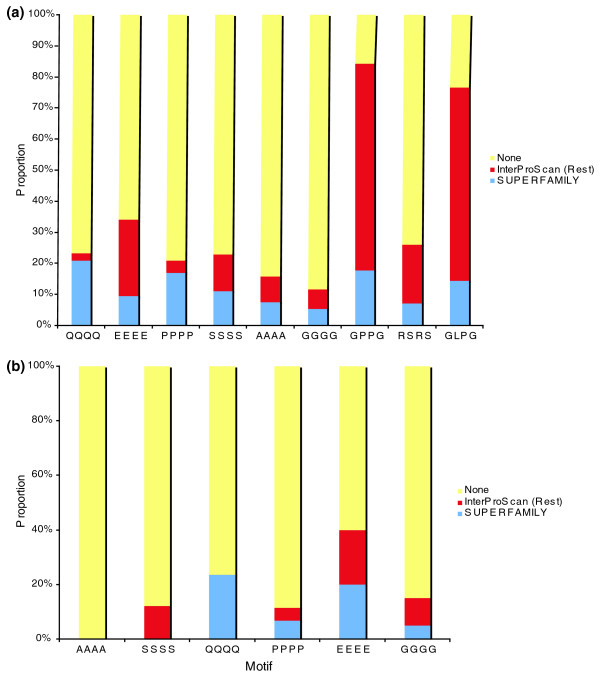
Proportions of AARs found within identifiable protein domains. **(a) **Tandem repeats; **(b) **C4 repeats. Repeats found within SUPERFAMILY domains are indicated by black bars. Additional repeats found within InterProScan domains are shown in grey and those outside domains by white bars. AARs are ordered by frequency.

To investigate the relative distribution of tandem and C4 repeats between structured and unstructured protein regions, we related the locations of repeats to protein domains, as defined by a search against the SUPERFAMILY [37] database (Figure 5a, b). SUPERFAMILY represents domains for which a three-dimensional structure is available and searches against it are, therefore, a stringent test for location of AARs within domains. Repeats were inferred to overlap domains if they lay entirely within the predicted domain. For tandem repeats the proportions of repeats lying within domains were between 10% for L and A and 20% for Q and E. For C4 repeats the range was between 0% for A_4 _and S_4 _and 24% for Q_4_.

These proportions represent a lower bound on the proportion of repeats lying within structured regions of proteins because structures have not been determined for all domains. An approximate upper bound can be estimated by considering the proportion lying within domains identified by InterProScan searches (excluding PANTHER; see Materials and methods). Many of these represent regions of proteins with functional associations but no known structure. Between 25% (for Q) and 95% (L) of tandem repeats lay within domains identified by InterProScan. Slightly lower proportions, between 0% (A_4_) and 40% (E_4_) of common homogeneous C4 repeats also lay within identifiable domains.

Tables 3 and 4 list the identifiable InterPro domains most commonly containing each of the main tandem and C4 repeat types. Of the tandem repeats, L repeats colocalized at high frequency with signal peptide domains identified by the SignalPHMM method [38]. Other tandem repeats showed less frequent associations with particular domains, although the domains they associated with in many cases are broadly consistent with their GO term associations. In particular, S and P repeats were most frequently found within protein-kinase-like domains. For C4 repeats few domains were found associated with repeats more than once. Notably, however, both E_4 _and P_4 _repeats were found associated more than once with the protein-kinase-like domain, mirroring results for S and P tandem repeats and consistent with the suggestion that some amino acid repeats are associated with cellular signaling cascades [32].

**Table 3:** Identifiable domains most frequently associated with tandem amino acid repeat types

Repeat type	Associated domain	Domain code	Number of hits	% of repeats
L	Signal peptide	signalp	111	55.2
S	Protein kinase-like (PK-like)	SSF56112	16	6.6
P	Protein kinase-like (PK-like)	SSF56112	11	3.8
Q	Quinoprotein alcohol dehydrogenase-like	SSF50998	5	3.6
A	Signal peptide	signalp	10	3.4
A	Transmembrane regions	tmhmm	10	3.4
E	WD40-repeat	SSF50978	9	3.0
G	Signal peptide	signalp	5	2.4

**Table 4:** Identifiable domains most frequently associated with cryptic amino acid repeat types

Repeat type	Associated domain	Domain code	Number of hits	% of repeats
QQQQ	Rm1C like cupin	SSF51182	4	23.5
EEEE	Protein kinase-like (PK-like)	SSF56112	3	12.0
SSSS	MYT1 (myelin transcription factor-like)	PF08474	2	6.1
PPPP	Protein kinase-like (PK-like)	SSF56112	2	4.5

We then considered the locations of tandem and C4 repeats compared to those of IURs. We predicted IURs using the RONN (Regional Order Neural Network) algorithm [39], which we selected because of its good performance, code accessibility and because it does not explicitly include information on the chemical properties of individual amino acids in its algorithm (although it may do so implicitly) - we preferred such a predictor as including chemical properties would introduce circularity into the analysis as we were investigating the propensity of particular chemical entities to lie within IURs.

**Figure 6:**
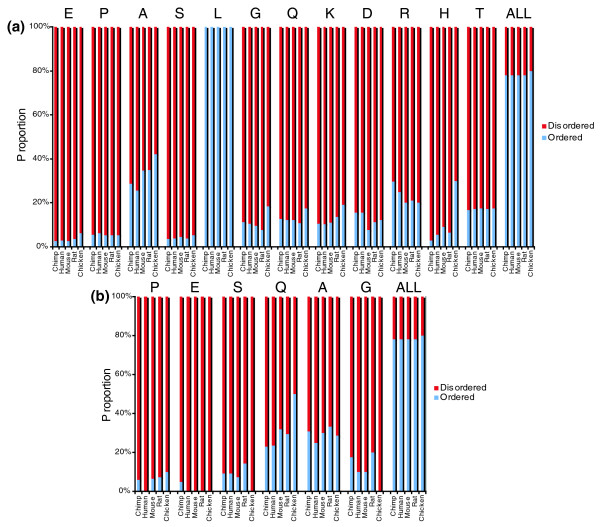
Proportions of AAR residues predicted to be ordered or disordered by RONN [39]. **(a) **tandem repeats; **(b) **C4 repeats. Ordered residues are shown in purple, disordered residues in red. Residues are ordered by frequency and frequencies for each proteome are presented in each group in the order: chimpanzee, human, mouse, rat, chicken. The section labeled 'ALL' in (a, b) shows the proportions of ordered and disordered amino acids in each proteome.

Residues with RONN scores of > 0.5 are predicted to be disordered (that is, IURs), whereas residues with scores < 0.5 are predicted to be ordered. Repeats were inferred to overlap IURs if they lay entirely within them. Figure 6 summarizes the proportions of amino acids within the different types of repeat that fall into the ordered and disordered classes across the five species.

Most repeats showed a strong tendency to lie in unstructured regions; for tandem repeats the proportions lying within unstructured regions ranged from 96% for E and S to 67% for A, compared to 22% for the average amino acid within a protein. The exceptions were L repeats, which were predicted to be predominantly ordered. Among C4 repeats, all the common repeat types again showed a strong preference for highly disordered regions. As for tandem repeats, E_4 _repeats showed the highest level of disorder while A_4 _showed a higher degree of order. Corresponding tandem and C4 repeats showed similar distributions between ordered and disordered regions. The exceptions to this trend were Gln repeats, which showed a higher tendency to be within structured regions as C4 repeats (32%) than as tandem repeats (13%).

Finally, we considered the proportion of IUR regions that contain an AAR. These proportions differ depending on the minimum length permitted for an IUR. For a minimum IUR length of 10, on average 85% of proteins contained a predicted IUR. Twenty to 21% of mammalian proteins and 13% of chicken proteins contained some kind of tandem AAR and 12% of mammalian proteins and 9% of chicken proteins contained some kind of C4 repeat; 4.6% of IURs contained a tandem AAR and 0.5% a C4 AAR. The proportion of proteins containing an IUR reported here is higher than the generally accepted proportion of around 40% [40,41]. We therefore investigated whether a longer length cut-off for our definition of an IUR would significantly affect these proportions. At a cut-off of 50 residues, 34% of proteins contain an IUR, which is similar to the proportion reported previously. Under this definition, 13% of IURs contained a tandem AAR and 2% a C4 AAR.

Numerous predictors of IURs are available - for a comparison see [42]. We compared results obtained with RONN to those obtained with two other predictors, DISOPRED [43,44] and IUPRED [45]. DISOPRED, like RONN, uses a machine learning approach coupled to protein structure information to predict IURs while IUPRED uses pairwise amino acid energy content. Comparison of the results from RONN with these predictors is shown in Table 5. Results from the three programs were broadly similar, with IUPRED and DISOPRED producing the closest result to RONN for an approximately equal number of tandem repeat types (four for IUPRED and three for DISOPRED). A notable difference was observed for A repeats, which were predicted as ordered in 63% of cases by IUPRED but 23% by DISOPRED and 32% by RONN.

**Table 5:** Comparison of predictions on locations of tandem repeats by three IUR predictors

Repeat type	Structured			Unstructured		
		
	RONN	DISOPRED	IUPRED	RONN	DISOPRED	IUPRED
E	3	13	**10**	97	87	90
P	6	5	**5**	94	95	95
A	32	**23**	63	68	77	37
S	4	**6**	16	96	94	84
L	100	97	**100**	0	3	0
G	11	**8**	14	89	92	86
Q	13	3	**14**	87	97	86

Values are the percentages of residues in the respective repeat types predicted to be unstructured or structured by the respective predictors. Values in bold are those more similar to the percentage predicted by RONN.

## Discussion

Although tandem repeats of amino acids are easily recognized features of proteins and have been extensively studied, protein sequences show more widespread repetitive features. This is shown by the high proportion of proteins containing repetitive segments - approximately 50% as measured by SEG [30] and over 70% of the *S. cerevisiae *proteome as measured by SIMPLE [32]. In this study we have compared the frequencies of tandem repeats with those of C4 repeats (repetitive regions with a local overrepresentation of motifs of length four residues) using SIMPLE, which has the advantage that it identifies explicitly the overrepresented motif in a given region. We have carried out this comparison in a large set of proteins orthologous between four mammals and chicken, which is the most closely related non-mammalian species with a sequenced genome. This allows us to compare repeat frequencies both between types and between species.

After excluding C4 motifs that overlap tandem repeats, many of the C4 motifs detected in these genomes are clearly related to common tandemly repeated amino acids (six of the seven most common tandem amino acid types in Figure 1a are mirrored by the six most common homogeneous C4 repeat types in Figure 1b), suggesting that the underlying mechanisms that gives rise to them is similar. This is also reflected in the high correlations seen between the frequencies of tandem repeats and their respective homogeneous C4 repeats. Tandem AARs most likely evolve by replication slippage, as they evolve more rapidly if they are encoded by pure codon repeats than interrupted codon repeats [6,13]. Dieringer and Schlötterer [46] introduced a novel, slippage-related process they called indel slippage that acts in a non-repeat-length-dependent manner on repeated motifs as short as a single nucleotide. Such a mechanism could contribute to the evolution of C4 repeats and other cryptically repetitive sequences [47,48] and could give rise to differences in the frequencies of tandem and cryptic repeats.

The biggest difference in frequency between tandem and cryptic repeats was seen for Leu, which is rare among C4 repeats. In addition, Q_4 _repeats are by far the most common class of C4 repeats while Gln is only the seventh most numerous class of tandem repeat in our sample. These large differences could reflect differences in underlying mechanisms (although this seems superficially unlikely as Q tandem repeats are known to undergo rapid evolution [6,49]) but could also reflect differential selective forces (acting strongly against L_4 _repeats and Q tandem repeats but less so against their counterparts).

Repeat frequencies were highly similar between the mammals, but the chicken proteome showed a distinct frequency distribution in which most repeat frequencies were lower. A partial exception to this pattern were tandem S repeats, which although rarer in chicken than in mammals, were the most common class in the chicken proteome. A trivial explanation for these differences could be the currently lower quality of the chicken genome sequence. However, this is unlikely to be the main explanation as the dataset we used contained only clearly identifiable orthologues. Another, and more interesting, possibility is that the lower frequency in chicken is the result of the general reduction of genome size in birds. The chicken genome is approximately one-third the size of the human genome [50] while bird genomes in general are approximately half the size of mammalian genomes [51]. Analysis of the evolution of bird genome size indicates that genome shrinkage took place in the saurischian lineage leading to the birds circa 200 to 300 million years ago and that this was accompanied by a reduction in the genome fraction of repetitive elements [51]. A global correlation of genome sequence repetition with genome size has also been described [52,53]. The lower frequency of amino acid repeats in chicken proteins may therefore reflect a parallel process of loss of transposable elements and tandem and cryptic repeats in that evolutionary lineage. A possible explanation for the stronger conservation of S repeats between mammals and chicken than other repeat types is that they play a less dispensable role in protein function; serine-rich domains (RS domains) are intimately involved in alternative splicing [54] and it is possible that this role is sufficiently important to ensure their retention.

Previous analyses of the evolution of Gln repeats have suggested that in the early stages of their emergence, when encoded by pure codon repeats, they appear preferentially in regions of proteins that are subject to relatively low levels of purifying selection (that is, regions that evolve more quickly than the rest of the protein) [7,21,22]. In this study we have analyzed the evolution of regions flanking tandem and C4 AARs in human-rodent and human-chicken comparisons and show the same trend, confirming that the majority of tandem and C4 repeats in proteins emerge in rapidly evolving subregions. We also confirm earlier suggestions [21,22] that conserved repeats lie in relatively more conserved protein subregions than non-conserved repeats and show that conserved AARs tend to lie in more conserved proteins than non-conserved AARs. In addition, we observe elevated sequence differences around conserved repeats of both types, although this elevation is less extreme than is observed for non-conserved repeats. The latter result differs from a previous study that did not find a difference between flanking regions and the remainder of the proteins for conserved AARs [21]. However, that study only considered a relatively small number of proteins and so most likely failed to detect this difference due to a lack of statistical power. Generally, the results are consistent with a model of repeat evolution whereby repeats tend to emerge in less-conserved regions of proteins and become frozen in length as they reach a length at which they are close to a threshold at which they may cause deleterious phenotypes [16,21] but they also suggest that the regions in which repeats become fixed may continue to evolve relatively rapidly after repeat fixation.

IURs are regions of proteins that do not form stable tertiary structures under native conditions. Analyses of the extent of disorder in whole genomes suggest that in eukaryotes more than 40% of proteins are either completely disordered or contain significant regions of disorder [40,41]. In this dataset we find 34% of proteins to contain IURs of length > 50 and 85% to contain an IUR of length > 10. These regions are thought to form flexible regions of proteins that might have a number of functions, including binding to other proteins and small molecules and providing flexibility in multidomain proteins. In an analysis of repeat content of a relatively small number of intrinsically unstructured protein regions, Tompa [25] identified an apparently strong role for AARs in IUR evolution. The definition of 'repeats' in his analysis is different from ours as it included longer, complex repeated motifs as well as simple sequence repeats, but some simple sequence repeats did appear in his results. This raises the question whether there is a real association of simple AARs with IURs, and whether an association of this type can account for the evolutionary dynamics of AARs. Here we have investigated this by considering the overlap between tandem and C4 repeats and, first, domains identifiable searching the SUPERFAMILY and InterPro databases, and second, unstructured regions predicted by the RONN predictor. The majority of AARs, with the exception of L tandem repeats, lie within IURs predicted by RONN (Figure 6).

We obtained inconsistent predictions on the level of structure shown by A repeats. They were predicted to be predominantly unstructured by two methods, RONN and DISOPRED, but not by a third, IUPRED. This disagreement may reflect the different methodologies employed by the different algorithms as IUPRED takes account of the chemical characteristics of the sequence being analyzed whereas RONN and DISOPRED use structural analyses of proteins. The ambiguous position of A in these analyses is interesting in the light of its role as the second major cause of human repeat expansion disease, after Q. Gln repeats are notable in showing markedly higher proportions of disorder as tandem repeats than as C4 repeats, suggesting that expansion of Q repeats could have a destabilizing effect on proteins, as suggested previously [18].

Seven of the eight most common tandemly repeated amino acids in our dataset correspond to the seven disorder-promoting amino acids defined by Dunker *et al*. [55]. Lise and Jones [56] in their study of common amino acid patterns in unstructured regions also identified a number of patterns similar to the most common C4 repeats, notably E- and P-rich regions. A strong element of the purifying selection acting against the emergence of AARs within folded regions of proteins therefore appears to be selection against their propensity to lower the stability of these regions. Interestingly, as noted by Kreil and Kreil [57], N repeats are much rarer than Q repeats - indeed, in our analysis of human proteins we found only four tandem N repeats. This observation may reflect the propensity of Asn to promote order [55] and consequent purifying selection acting against the appearance of N repeats in unstructured regions. A similar argument may apply to D and E repeats - Glu, which is common in AARs, is disorder-promoting whereas Asp, which is rare in AARs, is not. In this context, it is noteworthy that although E repeats are the most common class in mammals and the most often predicted to be unstructured, they are also, after L repeats, the class most commonly found associated with SUPERFAMILY and InterPro domains. This raises the question whether the domains in which they are located tend to be close to the threshold of instability. Mean RONN scores of domains containing E repeats are 0.44 for SUPERFAMILY and 0.46 for InterPro domains. These compare to means for all domains containing repeats of 0.43 for SUPERFAMILY domains and 0.41 for InterPro domains. The mean for E repeats in SUPERFAMILY domains is typical of all repeat-containing domains, but that for InterPro domains is the highest amongst all repeat types. As most of the domains containing E repeats are InterPro and not SUPERFAMILY domains, this raises the possibility that some E repeat-containing InterPro domains are relatively unstable.

L tandem repeats form interesting exceptions to the general association of AARs with unstructured regions as they are predicted to be 100% structured. The amino acids found in tandem repeats tend to be hydrophilic; all the most hydrophilic amino acids [58] are found in the class of common tandem AARs - the only strongly hydrophobic amino acid in this class is Leu. Hydrophobic amino acids tend to occupy buried positions within proteins, so it is not surprising that Leu repeats show a high propensity to be structured. In earlier analyses, Leu repeats have been found to be concentrated close to the amino termini of proteins [15,59], presumably forming part of the hydrophobic region of signal sequences, although Leu may also contribute to transmembrane segments of proteins and more generally to protein cores and stabilizing secondary and tertiary structure [59].

## Conclusions

The majority of AARs have arisen during evolution within protein regions with the characteristics of IURs. This is true both of tandem and cryptic repeats, which have many common characteristics such as relative frequency and, to a lesser extent, GO associations. The dynamics of the evolution of most AARs are, therefore, likely to mirror those of IURs. Some, but not all, IURs, evolve more rapidly than the proteins they are part of [27,28]. Despite this, our results suggest that only a small subset (no more than 15%) of IURs contain AARs. This raises the question whether there are specific subclasses of rapidly evolving IURs that have a higher propensity to evolve AARs. As AARs tend to be associated with transcription and cell signaling, it is possible that proteins with these types of functions have particular types of IUR that might predispose them to evolve repeats.

IURs are thought to play an important role in protein-protein interactions. Repeat accumulation may, therefore, play a role in the evolution of protein-protein interactions in transcriptional and signaling networks by expanding the repertoire of disordered regions. Because they evolve rapidly, repeat sequences potentially provide a means for organisms to rapidly tune their transcriptional and signaling protein-protein interaction networks [16].

Leu (and Ala) repeats form a special class in being hydrophobic amino acids that commonly form repeat structures. Leu repeats are consistently predicted to be structured, and Ala repeats often are. Glu repeats, which are very common, are also often found within structured regions, although Glu is disorder-promoting. Further studies of the evolution of these repeat classes are therefore merited as repeat variation in structured regions may be expected to have significant effects on protein structure and/or stability.

## Materials and methods

### Data set

For the analyses presented in this paper we prepared a set of orthologous proteins present in all five species, extracted from the Ensembl database version 41 [60]. We downloaded mouse, rat, chimp and chicken proteins that are orthologous to human proteins. All proteins were chosen to be orthologous to the same human protein. We excluded any duplicate entries, any sequences that were under 300 amino acids (thereby removing proteins too short to allow meaningful analysis of sequences' flanking repeats) and any human and mouse protein that did not have a Swissprot [61] identifier. The final dataset consisted of 5,815 orthologous proteins.

### Identification of amino acid repeats

Perfect tandem AARs were identified using a standalone JAVA program. Tandem repeats are defined here as continuous runs of a single amino acid with a length of more than four residues.

Cryptic repeats were identified using version 3 of the program SIMPLE [32] with modifications to increase its speed (S Greenaway, MS and JMH, unpublished [62]). To distinguish C4 repeats from overlapping tandem repeats, we excluded all C4 repeats that overlapped tandem repeats from further analysis. For any given repeat unit size, SIMPLE identifies sequence windows that achieve simplicity scores above any value seen in 100 randomized versions of the test sequence. The repeat unit corresponding to this window is recorded as a significantly simple motif (SSM). We considered repeats with repeat motifs of length four, which we call C4 repeats. For homogeneous motifs such as QQQQ (Q_4_), these correspond to regions that fall just below our definition of a tandem AAR. By looking at C4, rather than tandem repeats of a shorter length, we were also able to look at interrupted, tandem-like cryptic structures. It should be noted that using longer motif lengths would essentially replicate searches for tandem repeats of different lengths. Using shorter motif lengths (one to three) produces results more similar to those for tandem repeats than those seen for C4 repeats (data not shown).

### Evolutionary rate analysis

To confirm whether the flanking regions of AARs have evolved more rapidly than the whole protein, we constructed multiple alignments of orthologs from the five species using the default settings of CLUSTALW [63].

Replication slippage has been implicated as a mutational mechanism giving rise to variation in cryptically repetitive sequences [48] as well as being the major mutational mechanism at microsatellites [64]. As described previously for analyses of rates of evolution of sequences flanking microsatellites [49], care needs to be taken when analyzing the evolutionary rates of sequences flanking slippage-derived repetitive sequences. This is because sequences immediately flanking the repetitive sequence may also have been derived by slippage and subsequently modified by point mutation, and comparisons of these regions may, therefore, violate the requirement that aligned sites be homologous. By analogy with microsatellites at the DNA level, we therefore defined a transitional zone [49] for these analyses. This comprised all contiguous amino acid residues one mutational step away from the repeated motif at the codon level. For tandem repeats, the transitional zone started immediately amino- or carboxy-terminal to the limit of the repeat. For C4 repeats we took the region defined by the length of the window used to detect a significant motif (64 amino acids - that is, 30 amino acids either side of the central motif) to define the limits of the repeat, as this is the region containing a significant overrepresentation of the motif in question [32].

We then used Protdist from the PHYLIP package [65] to estimate the sequence divergence of a region 33 amino acids either side of the repeat plus transitional zone (the flanking region) and for the remainder of the protein less the flanking regions, transitional zone and repeat region [21]. Distance estimates calculated by Protdist were based upon the Jones-Taylor-Thornton model [66]. For regression analysis of flanking sequence of divergence against protein remainder divergence, outliers (regions whose residual divergence exceeded 2.326 standard deviations after accounting for the regression between flank and remainder) were removed from calculations.

### Gene Ontology term analysis

FatiGO+ [67] was used to identify level 3 and 4 GO terms significantly overrepresented in subsets of proteins containing particular repeat types. This analysis was carried out only on human and chicken proteins to minimize effects of multiple testing.

### Domain analysis

To test whether C4 and tandem repeats are embedded within functional domains or proteins, we searched for domains annotated in the Interpro database using the InterproScan web service [68-70]. The Interpro database characterizes a given protein, domain or functional site by integrating the most commonly used protein annotation databases. Hits to the SUPERFAMILY database were extracted from these results for separate analysis. Results from the PANTHER protein classification system were excluded from this analysis as they refer to protein function rather than domains [71].

### Prediction of intrinsically unstructured regions

IURs were predicted using the RONN algorithm [39]. Results from RONN were compared with two other predictors for which we could obtain code: IUPRED [45] and DISOPRED [43].

## Abbreviations

AAR: amino acid repeat; GO: Gene Ontology; IUR: intrinsically unstructured region; RONN: Regional Order Neural Network.

## Authors' contributions

MS carried out most of the data acquisition and analysis and drafted parts of the manuscript. JMH supervised the project, carried out some of the analysis, and compiled the final manuscript.
